# Balance and Health-Related Quality of Life After 1 Year of COVID-19 Social Restriction Measures: A Cross-Sectional Study in Two Samples from Spain

**DOI:** 10.3390/healthcare12212164

**Published:** 2024-10-30

**Authors:** Elisabet Huertas-Hoyas, Cristina Rodríguez-Rivas, Mª Pilar Rodríguez-Pérez, María García-de-Miguel, Nuria Trugeda-Pedrajo, Laura Delgado-Lobete, Gemma Fernández-Gómez, Lucía Rocío Camacho-Montaño

**Affiliations:** 1 Research Group in Evaluation and Assessment of Capacity, Functionality and Disability of Universidad Rey Juan Carlos (TO+IDI), Department of Physical Therapy, Occupational Therapy, Physical Medicine and Rehabilitation, Universidad Rey Juan Carlos, Avenida Atenas s/n, 28922 Alcorcón, Spain; elisabet.huertas@urjc.es (E.H.-H.); nuria.trugeda@urjc.es (N.T.-P.); gemma.fernandez@urjc.es (G.F.-G.); 2 Hospital Fundación Instituto San José, Avenida de la Hospitalidad, s/n, 28054 Madrid, Spain; c.rodriguezr.to@gmail.com; 3 Grupo 5, Centro Integral de Atención Neurorrehabilitadora, 28806 Zaragoza, Spain; mgm_96@hotmail.es; 4 Departament of Atención Sociosanitaria, Facultad de Ciencias Sociosanitarias, Universidad de Murcia, Avenida de las Fuerzas Armadas, s/n, 30800 Lorca, Spain; lauradelgado@um.es; 5 Unidad de Investigación en Cuidados de Salud (Investén-Isciii), Instituto de Salud Carlos III Avda, Pabellón 13, Monforte de Lemos, 28029 Madrid, Spain; luciacamachomont@gmail.com

**Keywords:** quality of life, occupational balance, pandemic, social constraints, home lockdown

## Abstract

Background: The COVID-19 pandemic significantly impacted the well-being of the general population. However, more information is needed regarding the relationship between participation-related outcomes. This study aimed to analyze the impact of the pandemic on occupational balance (OB) and health-related quality of life (HRQoL) before and after social restrictions and to explore their relationship with COVID-19 diagnosis. Methods: We conducted a study among individuals diagnosed with COVID-19, assigning a healthy control group with the same sociodemographic characteristics using the EQ-5D-5L and the Occupational Balance Questionnaire (OBQ). Results: The final sample size consisted of 61 participants in the COVID-19 diagnosis group (50.8% male; mean age 34.6 ± 14.17 years) and 57 healthy participants (50.8% male; mean age 33.7 ± 13.77 years). There were no differences in the sociodemographic variables between the groups. Significant differences were found between groups both before the pandemic and 1 year after confinement measures in HRQoL and OBQ (*p* < 0.005). The regression model indicated significant associations (*p* < 0.001) between HRQoL and both current OB and COVID-19 diagnosis. However, the OBQ scores from before the pandemic did not show a significant association with HRQoL (*p* = 0.336). Conclusions: In conclusion, social restrictions from the COVID-19 pandemic negatively impacted HRQoL in our sample even 1 year after confinement, with COVID-19 diagnosis and occupational imbalance predicting worse outcomes, highlighting the need for targeted interventions not only for the current situation but also for possible future public health crises.

## 1. Introduction

COVID-19, which was declared a global pandemic in March 2020, has been shown [1,2] by research evidence to lead to several physical risks affecting health-related quality of life (HRQoL) due to its various symptoms [3,4,5,6]. Similarly, mental health-related outcomes resulting from COVID-19 significantly impair patients’ quality of life (QoL) and may affect their daily performance and health-related well-being [7]. In Spain, several social measures were implemented in order to prevent disease spread, such as home lockdowns, age-related restrictions on leaving home, social distance between persons, closing time restrictions in restaurants, night curfew, and restraint measures on commercial activity and cultural facilities or limits on the mobility between regions. Any infraction of these measures was sanctioned. In addition, in-person health care provision was mostly replaced by telehealth systems. All these measures were at least partially maintained until 2021. Currently, Spanish citizens are not still required to submit to restrictions anymore, but the incidence of COVID cases is increasing, so it would not be surprising if certain mild restrictions were reinstated, such as wearing masks on public transport and specific indoor placements.

Among these measures, home lockdown indirectly had a great impact on daily activities, habits, and routines [8,9,10], negatively affecting life satisfaction [11]. The WHO has warned about the potential psychosocial consequences triggered by the self-isolation of the pandemic, feelings of uncertainty, and the disruption of daily routines. Specifically, studies have reported that changes in work patterns and daily life are increasingly associated with psychological symptoms and burnout during the pandemic. It is crucial to understand how the disruption of daily activities during the pandemic has affected current health, with a view to other possible crisis scenarios [12]. Similarly, studies have reported a negative effect on mental health, as shown by the alarming increase in anxiety, depression, and post-traumatic stress disorder symptoms in the general population [13]. This is of great importance, as high levels of stress and anxiety affect HRQoL, and moderate/high levels of stress have been negatively associated with HRQoL [11,14,15].

Occupational balance (OB) refers to the ability of individuals to participate in a variety of meaningful daily activities in a manner that aligns with personal needs, values, and time management. It involves achieving a harmonious distribution of work, leisure, self-care, and rest. OB is considered essential for maintaining overall health and well-being, as imbalances in these areas can lead to physical and mental health issues, such as stress and decreased life satisfaction. Previous studies have shown that balanced participation in satisfying occupations contributes to the maintenance of health and well-being, while occupational imbalance is associated with higher levels of internalizing problems, such as stress, which consequently has a negative impact on a person’s overall health [16,17]. The relationship between occupational balance, health, QoL, and life satisfaction has been previously studied [18]. It has been shown that performance in an adequate range of occupations in daily life is an essential need, even when it is necessary to adapt such performance to new situations [19]. Occupational balance involves not only participating in a variety of daily activities but also being able to manage time and distribute such activities in a healthy and productive manner, which is strongly associated with personal autonomy (parks-31) [20]. In this study, occupational balance is particularly relevant due to the significant disruptions caused by the COVID-19 pandemic, which altered daily routines and limited participation in various activities. These changes likely impacted the population’s ability to maintain occupational balance, making it crucial to explore its relationship with health-related quality of life (HRQoL) during and after the period of social restrictions.

Some authors, including González-Bernal et al. [19], have concluded that there is a significant correlation between occupational balance (OB) and physical and mental health during confinement. On the other hand, López-Moreno et al. (2020) [21] carried out a study to explore the impact of the COVID-19-derivated home lockdown on the lifestyles and emotional balance of the Spanish population. After analyzing the data of 675 Spaniards, they concluded that almost 40% of the sample reported problems regarding sleeping habits and sleeping quality; moreover, almost half of the sample did not perform physical activity. Overall, all these significant and sudden changes in the daily routine may lead to a decreased QoL. However, research on this topic is scarce, and the contribution of occupational balance to QoL and whether a COVID-19 diagnosis can exacerbate occupational balance and QoL remains unclear.

To the best of our knowledge, no study has explored the impact of COVID-19-related restrictions 1 year after their enactment on balance and HRQoL in the Spanish population for the period during which rigorous restriction measures were being implemented [22,23,24,25,26,27]; there are reports of a decrease in QoL during home confinement. However, there is no data regarding whether these impacts diminished once home confinement was lifted and replaced by other social isolation measures.

Moreover, the measures of social restriction have varied according to whether the risk of spread increased during the last 2 years. In addition, no study has analyzed the alterations in OB and QoL and its relationship after a year of social restrictions.

This is of special relevance in order to understand the scope of the social restriction policies during the COVID-19 pandemic on the QoL, which, in turn, may contribute to the design of individualized intervention strategies that take into account those aspects that may be associated with lower QoL, such as occupational balance. It is crucial to understand how the disruption of daily activities during the pandemic has affected QoL and to comprehend how the imbalance between different occupational areas can exacerbate the effects of COVID-19 pandemic restriction measures, not only for the current situation but also as an important factor for future public health crises and other possible crisis scenarios [12].

The hypothesis of this study was that decreased QoL caused by social isolation would persist even after the most restrictive social isolation measures were lifted and that this would be partially explained by the impact of social isolation on Occupational Balance.

The main objective of this study was to analyze the impact of the COVID-19 pandemic on HRQoL in Spanish adults 1 year after the declaration of the pandemic. The secondary objectives were the following: (1) to analyze the differences between COVID-19 diagnosis and the healthy subsample; (2) to explore the relationship between HRQoL and Occupational Balance; (3) to investigate the relationship between HRQoL as the dependent variable and COVID-19 diagnosis, with pre-OBQ and post-OBQ as the independent variables.

## 2. Materials and Methods

### 2.1. Design

We conducted a descriptive, cross-sectional study following the guidelines for observational studies (STROBE) [28]. This study was secondary to the work published by Rodriguez-Rivas et al. (2022) [29]. The protocol was approved by the King Juan Carlos University Ethics Committee (reference number 1507202117221). All participants were informed in advance about the details of the research. In compliance with the Declaration of Helsinki, informed consent was requested [30].

### 2.2. Participants

A non-probabilistic convenience sampling method was used to recruit participants through a snowball technique. The sample of volunteers was recruited between 19 February and 8 March 2021 via social media (i.e., Facebook and WhatsApp). The exposure variable was the diagnosis of COVID-19. The sample was subdivided into patients after diagnosis and healthy controls, using the same sociodemographic characteristics.

The inclusion criteria for the participants were (1) men or women aged 18 years or older, (2) currently living in Spain, and (3) signed an informed consent form. The exclusion criteria were (1) an inability to respond to the outcome measures and (2) the presence of other diseases arising after February 2020 except COVID-19 diagnosis.

### 2.3. Data Collection

An online survey was designed and created using the Office365 Microsoft Forms application (Microsoft Office 365). The questionnaire collected health-related and sociodemographic data (i.e., sex, age, region of residence, and COVID-19 diagnosis). The latter two variables were measured using standardized tools. The EQ-5D-5L questionnaire was used to assess health-related quality of life (HRQoL), and the Occupational Balance Questionnaire (OBQ) was used to measure OB.

The participants were asked to inform about their current HRQoL and OB (February to March 2021) and their HRQoL and OB when the social restrictions were mandatory (March 2020).

### 2.4. Procedure

Once the questionnaire was developed, it was distributed through social media, including WhatsApp. Potential participants were able to get information about the study aims and methods, and only after providing informed consent were they redirected to the online questionnaire. Data underwent a codification process in order to ensure the participants’ anonymity and were only available to the main researcher.

### 2.5. Measures

#### 2.5.1. EuroQol 5D–5L (EQ-5D-5L)

HRQoL was assessed using the EQ-5D-5L questionnaire, which includes five dimensions (mobility, self-care, activities of daily living, pain/discomfort, and anxiety/depression), each scored on a five-point ordinal scale (1 = “no problems”, 2 = “mild problems”, 3 = “moderate problems”, 4 = “severe problems”, and 5 = “extreme problems”). Higher scores on the scale indicate a better quality of life (QoL). None of the items were reverse-coded before scaling. The results were calculated using the tool-specific software [31]. The EQ-5D-5L is currently available in more than 130 languages. Based on the study carried out by Feng et al. [32], excellent psychometric properties in similar populations, with adequate internal consistency and robust validity, have been demonstrated. Their results overall solidly establish the validity of the EQ-5D-5L, as supported by observed trends across subgroups (pooled means and known-group validity), as well as convergent validity (correlation of items and index to other measures of health-related quality of life).

#### 2.5.2. Occupational Balance Questionnaire (OBQ)

The OBQ is a questionnaire that assesses the perceived OB in relation to someone’s daily life. It measures the ability to manage the amount and variability of tasks within an occupation while preserving personal preferences, as well as the ability to maintain a strong sense of self-identity through participation in meaningful occupations based on personal values [33]. The OBQ particularly focuses on a person’s satisfaction with the range and variability of occupations and provides a global picture of one’s own occupational balance [34]. It consists of 13 items exploring OB, meaning, time use, and satisfaction. It is scored using a six-point Likert scale, with values ranging from 0 (“strongly disagree”) to 5 (“strongly agree”). The total score ranges between 0–65, in which a higher score indicates better OB. The OBQ has been adapted and validated for Spanish population (OBQ-E), Psychometric testing showed excellent internal consistency (Cronbach’s alpha = 0.87; Guttman split-half coefficient = 0.85), good test-retest reliability (rs (Spearman rho) = 0.73), and acceptable convergent validity (overall status, rs = 0.37; Related Quality of Life, rs = 0.42; Satisfaction with Life Scale, rs = 0.54). Validation measures of the final version of the OBQ-E were conducted in a sample of 219 participants. The OBQ-E showed that items and instructions were culturally appropriate and written clearly [33].

### 2.6. Data Analysis

IBM SPSS Statistics 29.0 (IBM SPSS Corp; Armonk, NY, USA). and RStudio were used for the statistical analyses. The frequency of categorical variables and the mean and standard deviation of numerical variables were calculated. Differences across sociodemographic variables in each group were examined using Student’s *t*-test for independent samples for numerical variables, and the chi-square test was applied for nominal variables.

RStudio was used to plot the scores of the EuroQoL 5D-5L and OBQ from before the pandemic to 1 year after the confinement. Differences between pre- and post-social restrictions HRQoL and OBQ were calculated using the paired samples *t*-test in each group. Additionally, the Student’s *t*-test for independent samples was applied to examine differences between groups. Pearson’s correlation coefficient was used to look at correlations between HRQoL and OB, and RStudio was also utilized to visualize the correlations according to the COVID diagnosis. A multivariate regression model was developed to identify possible predictors of HRQoL. In this model, HRQoL was the dependent variable, while pre-OBQ (before the pandemic), post-OBQ (current), and COVID-19 diagnosis were independent variables. RStudio was also used to correlate the variables that were to be introduced into the regression model to address multicollinearity issues. In addition, the variance inflation factor test was used for multicollinearity analyses. The overall model fit equation and power analysis were included as part of the multiple regression model.

## 3. Results

The final sample consisted of 118 participants, divided into two groups: the sample that had been diagnosed with COVID-19 (50.8% male and mean of age 34.6 ± 14.17 years) and the healthy sample (50.8% male and mean of age 33.7 ± 13.77 years). The descriptive data of the sample can be found in Table 1, along with the tests (Student’s *t*-test for independent samples, Chi-square test, or Fisher’s Exact Test, as applicable depending on the type of variable), which show that there are no differences between groups in the sociodemographic variables. As the results demonstrate, the two groups were homogeneous in terms of sociodemographic variables.

**Figure 1:**
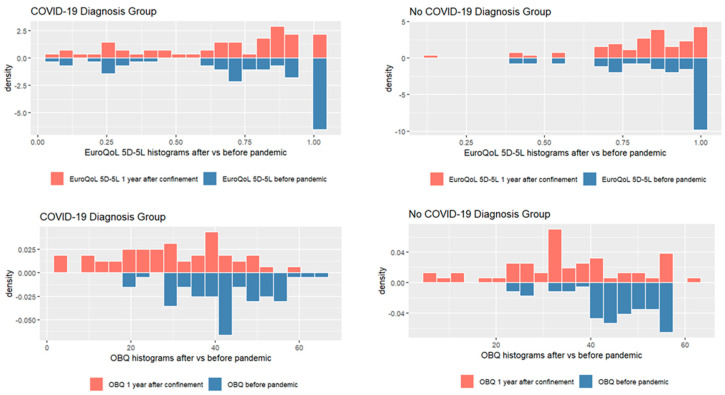
EuroQoL 5D-5L and OBQ histograms after vs. before the pandemic.

Figure 1 shows that the HRQoL scores, measured by the EuroQoL 5D-5L, decreased in both samples from before the pandemic to 1 year after confinement. The scores in occupational balance, measured by the OBQ, also decreased compared to before the pandemic, indicating a higher occupational imbalance after 1 year of social restriction measures. See Supplementary File S1. Descriptive analysis of QOL and OB scores.

Table 2 shows that HRQoL, measured by the EuroQoL 5D-5L, significantly decreased in both groups from before the pandemic to 1 year after confinement, according to paired samples *t*-tests, resulting in a lower perceived HRQoL. When comparing independent samples based on COVID-19 diagnosis, there were significant differences between the groups both before the pandemic and 1 year after confinement measures.

The scores in occupational balance, measured by the OBQ, significantly decreased in both groups compared to before the pandemic, indicating higher occupational imbalance in both groups after 1 year of social restriction measures. There were significant differences between the groups both before the pandemic and 1 year after confinement (Table 2).

**Figure 2:**
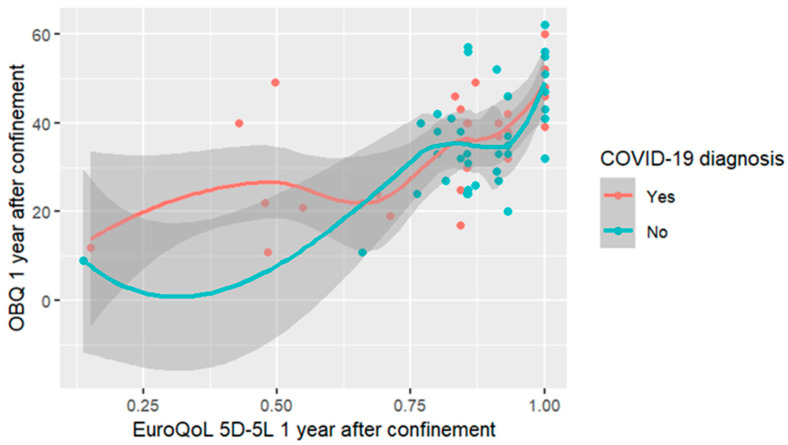
Correlation between OBQ and HRQoL according to COVID-19 diagnosis.

Moderate and significant correlations were found between OBQ and EQ-5D-5L scores in both samples. Figure 2 shows that higher OB was associated with better HRQoL 1 year after the social restrictions period in the COVID-19 diagnosis sample (r = 0.644, *p* < 0.01) vs. no COVID-19 diagnosis (r = 0.532, *p* < 0.001).

A multicollinearity test was conducted to identify any relationships between the independent variables that might lead to multicollinearity before performing the regression analysis. The results assessed how different variables (COVID-19 diagnosis, pre-OBQ, and post-OBQ) relate to the outcome, EuroQoL, during the pandemic. In this case, no multicollinearity issues were found for any of the independent variables (see Supplementary File S2).

According to the multiple regression model, HRQoL was significantly associated with OB and COVID-19 diagnosis, which, overall, explained over 40.5% of the variance of HRQoL (F = 21.745; adjusted R^2^ = 0.386; *p* < 0.001). The model shows a significant association of HRQoL with current OB and COVID-19 diagnosis. Specific possible predictors of HRQoL, such as current OBQ and COVID-19 diagnosis, have significant *t*-test results (*p* < 0.001), while OBQ before the pandemic does not show a significant association (*p* = 0.336). The standardized beta is higher for occupational balance after the lockdown than for the COVID-19 diagnosis (Table 3).

Based on the provided results, the overall model fit equation can be expressed as follows:

$$EuroQoL_{i}∼N\left({\hat{\mu }}_{i},{\hat{\sigma }}^{2}\right)$$

μi=0.239+0.086⋅Covid19i+0.009⋅OBQposti+0.002⋅OBQprei

and the residual standard error is $\hat{\sigma }=0.1842$.

A power analysis was conducted using the three independent variables as predictors, with the effect size calculated as $\frac{R^{2}}{1-R^{2}}$, a significance level of 0.05, and a power of 0.8. The result yielded an f2 of 0.68 and degrees of freedom of 16.41.

## 4. Discussion

The main purpose of this study was to examine the impact of restrictions implemented during the COVID-19 pandemic 1 year after its declaration on HRQoL and its relationship with occupational balance in the Spanish population across both COVID-19 and non-COVID-19 groups and to investigate the potential impact of a COVID-19 diagnosis.

The findings show a significant decrease in the level of HRQoL compared to pre-pandemic HRQoL in both groups. In line with this, previous studies conducted in different countries have found poorer QoL during confinement [24,25,26,27,29]. For instance, Lipskaya-Velikovsky (2021) [25] described lower QoL in the physical, psychological, and social relations domains during home confinement in the healthy population of Israel.

It should be noted that people with a positive COVID-19 diagnosis reported significantly lower HRQoL than the healthy population. One possible explanation for this outcome is that people who had COVID-19 might also have had previous health problems, such as chronic diseases or other health conditions, which would contribute to both COVID-19 contagion and poorer QoL, as suggested by Nguyen et al. (2020) [35,36]. Moreover, as Mitrović-Ajtić et al. (2022) [37] indicate, the consequences of COVID-19 may persist even after 5 months or be replaced by persistent COVID-19. For instance, reductions in mobility were three to four times more likely in elderly post-COVID-19 patients, whose levels of pain and discomfort increased. Nevertheless, there is a lack of research focused on these differences; therefore, additional studies are needed to analyze and test these hypotheses. Specifically, Chen et al. (2020) [38] explored the differences in QoL between patients with COVID-19 and the general population using the Short-Form (SF-36) Health Survey. They found that COVID-19 patients reported significantly lower HRQoL than healthy controls in all domains except for physical function. In accordance with our results, they found significant differences in HRQoL between those with and without a COVID-19 diagnosis. Future studies may expand on these aspects, as our findings show that prolonged social isolation after 1 year can negatively impact health and quality of life in both the general population and individuals affected by COVID-19 symptoms.

The data reveal that there is a relationship between OBQ and HRQoL variables, which is stronger in the COVID-19 group. The regression model indicates that COVID-19 diagnosis and OBQ during the pandemic (1 year after confinement) together explained over 40.5% of the variance in HRQoL among Spanish adults, excluding occupational balance before the pandemic, which was not significant in the regression model. According to the standardized beta value in our regression model, occupational balance after the lockdown indicated a greater influence on quality of life than the COVID-19 diagnosis. Our results also found negative effects on occupational balance, being worse in the group with a COVID-19 diagnosis. A previous longitudinal study [12] analyzing occupational balance found that the more sports and social contact people had, the lower they scored in depressive symptoms. However, the effect faded or disappeared by wave 4 of the lockdown, possibly due to the impact of the final lockdown on mental health, making protective activities such as sports and social contact less effective. This seems a plausible hypothesis, particularly after months of restrictions that have led to exhaustion in the population. It seems that our data from the regression model support this hypothesis. It appears that occupational balance 1 year after the lockdown significantly impacts quality of life, unlike the balance before the pandemic.

These results could suggest that intrinsically restrictive COVID-19 measures, by disrupting occupational balance, lead to a poorer quality of life. In this line, previous authors [24] have found that the number of interrupted activities and dissatisfaction with participation during confinement were significant contributing variables to HRQoL using the World Health Organization Quality of Life Instrument (WHOQOL-BREF). Thus, our results suggest that these factors continue to influence HRQoL if social constraints are maintained. Based on the results of Park et al. (2019) [20], there is a relationship between OB, QoL, and health-related variables, with a positive effect on the health of those with the highest levels of OB. In the study of Håkansson et al. (2021) [39], it was shown that those with better OB did not show stress, concluding that occupational imbalance was associated with greater stress. In turn, those with higher OBQ scores were those who had better work support conditions, participated in various leisure activities and hobbies, and spent more time resting. Thus, it may be concluded that deficits in OB contribute to a worse mental health-related QoL. However, we have not found longitudinal studies that analyze occupational balance and its relationship with quality of life.

The findings of this study emphasize the importance of maintaining a structured occupational balance to support overall well-being, even during a pandemic, and especially in restarting routines after the pandemic.

### Limitations, Future Research, and Professional Implications

Our study has some limitations that need to be addressed. First, the sample size in each group may not be sufficient to generalize our results. Self-reported data collection may lead to bias; however, to avoid response bias and social desirability bias, the questionnaires were anonymous. This methodology has been used in previous similar studies [22,39]. Additionally, there may be biases associated with using online surveys for data collection, such as limited access or technological proficiency in certain age groups. Second, there may be sample selection bias, as the number of participants aged 36–64 years was smaller. This may be explained by the recruitment method, as adults in this age group may use new technologies to a lesser extent and have more difficulty using them. To address these potential biases, we employed strategies such as non-probabilistic sampling and the use of a broad sample to enhance representativeness. Future longitudinal studies on the impact of longer-term confinement are needed. Moreover, it would have been interesting to add a retrospective assessment of pre-COVID-19 HRQoL. The severity of the symptoms and the duration of the COVID-19 illness were not controlled.

However, our study has several relevant implications. The situation experienced during the pandemic provided an overall restrictive context suitable for this study, which offers valuable information on the impact of social isolation that may be useful for improving therapeutic treatments involving long periods of social interaction restriction (e.g., people with immune deficiencies). Additionally, our findings regarding the contribution of OB to HRQoL in a socially restrictive context may be used by mental health practitioners. Occupational imbalance should be addressed in those experiencing social isolation. The significant association between OBQ and HRQoL 1 year after confinement suggests the need for interventions aimed at preserving or restoring occupational balance to mitigate the negative impact on quality of life caused by COVID-19-related disruptions. Given the importance of occupational balance and health-related quality of life in situations of prolonged social restrictions, future studies should explore the development of targeted interventions designed to restore or maintain occupational balance, particularly in vulnerable populations such as individuals with chronic health conditions or those at higher risk of social isolation. Longitudinal studies are especially needed to examine the long-term impact of social restrictions on occupational balance and health outcomes, not only in the context of the current pandemic but also for future public health crises.

From a professional perspective, our results underscore the critical need for targeted interventions, not only during the current pandemic but also in preparation for possible future public health crises. Effective public health strategies and support systems can help mitigate long-term adverse effects on quality of life and ensure better health outcomes in the face of similar challenges. Health practitioners, especially those in occupational therapy and mental health, should develop tailored interventions that address the disruptions in daily routines caused by social isolation, focusing on promoting occupational balance as a key factor in mitigating negative effects on quality of life and well-being. Moreover, the role of digital tools in ensuring equitable access to healthcare and social support during crises should be further explored to promote participation across all demographic groups.

This underscores the need for further longitudinal studies to analyze this impact over an extended follow-up period. It highlights the critical need for targeted interventions, not only during the current pandemic but also in preparation for possible future public health crises. Effective public health strategies and support systems can help mitigate long-term adverse effects on quality of life and ensure better health outcomes in the face of similar challenges.

## 5. Conclusions

This study demonstrates the negative impact of social restrictions resulting from the COVID-19 pandemic on HRQoL and explores the role of OB after 1 year of social isolation. COVID-19 diagnosis and occupational imbalance appear to predict worse current HRQoL outcomes. These findings underscore the importance of preserving or restoring occupational balance to mitigate the negative impact on quality of life caused by COVID-19-related disruptions. More longitudinal studies are needed to understand these effects over time, emphasizing the critical need for strategies that support long-term health outcomes, not only for the current situation but also for potential future public health crises.

## Figures and Tables

**Table 1:** The sociodemographic characteristics of the samples.

Variables	COVID-19 Diagnosis (n = 61)	No COVID-19 Diagnosis (n = 57)	*X*/*t* *	*df*	*p*
Sex (n [%]) Male Female	31 (50.8%) 30 (49.2%)	28 (49.1%) 29 (50.9%)	0	1	1
Age (M [SD]) Years	34.60 (14.174)	33.68 (13.768)	0.409		0.342
Age range (n [%]) 18–35 years 36–64 years	39 (63.9%) 22 (36.1%)	37 (64.9%) 20 (35.1%)	0	1	1
Region of residence (n [%]) North Centre South	5 (8.3%) 52 (86.7%) 3 (5%)	5 (8.9%) 48 (85.7%) 3 (5.4%)	0.022	NA **	1

Notes: M, mean; SD, standard deviation; *t*: Student’s *t*-test for independent samples; *X*: *X*-squared test; *df*: degrees of freedom; *p*: *p* value for test. * In nominal variables, *X* is calculated, and in numerical variables, *t*. ** NA: Not applicable; in the case of place of residence, the *X* test was invalid, so it was replaced by Fisher’s exact test (some of the subgroups have small sample sizes) for which the results were *p* = 1 with an alternative hypothesis of two-sided.

**Table 2:** HRQoL and OBQ before and 1 year after confinement and other related COVID-19 restrictions.

	COVID-19 Diagnosis (n = 61)			No COVID-19 Diagnosis (n = 57)			*t*	*p*
	M ± SD	*t*	*p*	M ± SD	*t*	*p*		
**EuroQoL 5D-5L** Before pandemic 1 year after confinement	0.73 ± 0.294 0.65 ± 0.281	3.352	0.001	0.86 ± 0.175 0.81 ± 0.176	2.541	<0.001	2.783 3.472	**0.003** **<0.001**
**OBQ** Before pandemic 1 year after confinement	43.98 ± 10.706 29.60 ± 13.913	5.925	<0.001	45.28 ± 9.168 34.94 ± 13.581	4.941	0.007	1.968 1.683	0.026 0.048

Notes: M, mean; SD, standard deviation; *p*-values were calculated using the paired samples *t*-test; *p*-values in bold were calculated using the independent samples *t*-test.

**Table 3:** Regression model summary (n = 118).

R	R^2^	R^2^ Adjusted	Standard Error of Estimation	F-Test	*p*	Durbin-Watson	Independent Variables	(95% CI) ^b^	Std. ^b^	*t*-Test	*p*
0.636 ^a^	0.405	0.386	0.184	21.745	0.001	1.739	(Constant) **Post-OBQ** **Pre-OBQ** **COVID-19 diagnosis**	0.239 (0.032, 0.445) 0.009 (0.007, 0.012) 0.002 (−0.002, 0.005) 0.086 (0.011, 0.160)	0 0.562 0.072 0.183	2.297 6.965 0.909 2.283	0.024 <0.001 0.336 <0.001

Notes: ^a^ independent variables; ^b^ dependent variable: EuroQol 5D-5L during pandemic; OBQ, occupational balance questionnaire. Bold values are statistically significant. b (95% CI).

## Data Availability

Data is contained within the article and Supplementary Material.

## Supplementary Materials

The following supporting information can be downloaded at: https://www.mdpi.com/article/10.3390/healthcare12212164/s1. Figure S1. Correlations between variables introduced in the regression model; Table S1. Descriptive analysis of QOL and OB scores for the total sample before and after the pandemic; Table S2. Multicollinearity test based on the regression model.
