# Supplementary material for: Balance and Health-Related Quality of Life After 1 Year of COVID-19 Social Restriction Measures: A Cross-Sectional Study in Two Samples from Spain
Source: Healthcare (Basel). 2024 Oct 30;12(21):2164. doi: 10.3390/healthcare12212164 (PMC11545742; doi:10.3390/healthcare12212164)
Supplement: Supplementary file 1 [file healthcare-12-02164-s001.zip › healthcare-3220172-supplementary.pdf]

healthcare

MDPI

Supplementary File S1. Descriptive analysis of QOL and OB scores.

Table S1. Descriptive analysis of QOL and OB scores for the total sample before and after the pandemic.

	COVID-19 diagnosis (n=61)	No COVID-19 diagnosis (n =57)	Total sample (n=118)
	M±SD	M±SD	M±SD
EuroQoL 5D-5L<br>*Before pandemic*<br>*1 year after confinement*	 0.73±0.294<br>0.65±0.281	 0.86±0.175<br>0.81± 0.176	 0.91±.248<br>0.79±.246
OBQ<br>*Before pandemic*<br>*1 year after confinement*	 43.98±10.706<br>29.60±13.913	 45.28±9.168<br>34.94±13.581	 43.75±9.98<br>32.38±13.86

Supplementary File S2. Multicollinearity test

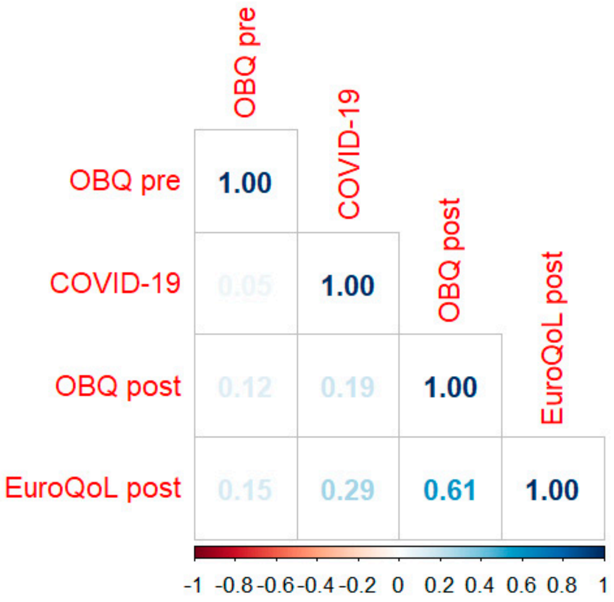

Figure S1. Correlations between variables introduced in the regression model.

Table S2. Multicollinearity test based on the regression model.

Covid19 diagnosis	OBQ pre	OBQ post
**1.037783**	**1.015277**	**1.050118**
